# Chronic Low Back Pain in Young Adults: Pathophysiological Aspects of Neuroinflammation and Degeneration

**DOI:** 10.3390/ijms26157592

**Published:** 2025-08-06

**Authors:** Natalya G. Pravdyuk, Anastasiia A. Buianova, Anna V. Novikova, Alesya A. Klimenko, Mikhail A. Ignatyuk, Liubov A. Malykhina, Olga I. Patsap, Dmitrii A. Atiakshin, Vitaliy T. Timofeev, Nadezhda A. Shostak

**Affiliations:** 1 Acad. A. I. Nesterov Department of Faculty Therapy, Pirogov Russian National Research Medical University, 1, Ostrovityanova St., 117997 Moscow, Russia; annove2008@mail.ru (A.V.N.); aaklimenko@yandex.ru (A.A.K.); nshostak44@mail.ru (N.A.S.); 2 Research and Educational Resource Centre for Immunophenotyping, Digital Spatial Profiling and Ultrastructural Analysis Innovative Technologies, Patrice Lumumba Peoples’ Friendship University of Russia (RUDN University), 6, Miklukho-Maklaya St., 117198 Moscow, Russia; anastasiiabuianova97@gmail.com (A.A.B.); ignatyuk-ma@rudn.ru (M.A.I.); malyhina-la@rudn.ru (L.A.M.); olga@patsap.ru (O.I.P.); atyakshin-da@rudn.ru (D.A.A.); 3 Scientific Research Laboratory of Rheumatic Disease, Pirogov Russian National Research Medical University, 1, Ostrovityanova St., 117997 Moscow, Russia; vtt3@yandex.ru

**Keywords:** back pain, degenerative disc disease, aggrecan, NGF, substance P, S-100, calcification, immunohistochemistry, young age

## Abstract

Degenerative disc disease (DDD) is a major cause of chronic low back pain (LBP), yet the molecular mechanisms driving disc degeneration and pain remain poorly understood. This study analyzed intervertebral disc (IVD) tissue from 36 young patients (median age = 36.00 [31.00, 42.50] years) with herniated discs and LBP, alongside healthy controls, to investigate changes in the extracellular matrix (ECM) and neurochemical alterations. Disc degeneration was assessed using MRI (Pfirrmann grading) and histology (Sive’s criteria). Histochemical and immunohistochemical methods were used to evaluate aggrecan content, calcification, and the expression of nerve growth factor (NGF), substance P (SP), and S-100 protein. MRI findings included Pfirrmann grades V (30.55%), IV (61.11%), III (5.56%), and II (2.78%). Severe histological degeneration (10–12 points) was observed in three patients. Aggrecan depletion correlated with longer pain duration (r = 0.449, *p* = 0.031). NGF expression was significantly elevated in degenerated discs (*p* = 0.0287) and strongly correlated with SP (r = 0.785, *p* = 5.268 × 10^−9^). Free nerve endings were identified in 5 cases. ECM calcification, present in 36.1% of patients, was significantly associated with radiculopathy (r = 0.664, *p* = 0.005). The observed co-localization of NGF and SP suggests a synergistic role in pain development. These results indicate that in young individuals, aggrecan loss, neurochemical imbalance, and ECM calcification are key contributors to DDD and chronic LBP.

## 1. Introduction

Low back pain (LBP) is one of the leading causes of disability and work incapacity worldwide, affescting individuals across all age groups [1,2]. In recent years, a rising incidence among young adults has made LBP an escalating public health concern. By 2050, the global number of individuals affected by LBP is projected to increase by 36.4% compared to 2020, reaching approximately 843 million cases [3]. Among the various etiologies of LBP, intervertebral disc (IVD) pathology accounts for more than half of all cases [4].

The clinical condition characterized by back pain alongside imaging-confirmed disc degeneration is referred to as degenerative disc disease (DDD) [5]. Hallmark features of DDD include dehydration of the disc, degradation of extracellular matrix (ECM) components, macroscopic cartilage defects, varying degrees of disc herniation or extrusion, neurovascular ingrowth, and, in some cases, calcification [6].

The IVD comprises three anatomically and functionally distinct components: the highly hydrated, proteoglycan-rich nucleus pulposus (NP); the annulus fibrosus (AF); and the cartilaginous endplates (EPs), which anchor the disc to adjacent vertebral bodies [7]. In healthy individuals, NP hydration ranges between 70–90%, with its structural matrix consisting of 35–65% proteoglycans, 5–20% type II collagen, and small amounts of elastin and other proteins [8]. The NP contains large notochordal-derived cells and mesenchymal cells with chondrocyte-like morphology, which, during maturation and degeneration, transition into smaller fibrochondrocytes nested within lacunae [9,10]. The AF, by contrast, contains approximately 65–70% water, 20% proteoglycans, 50–70% type I collagen, and around 2% elastin. Together, these components contribute to the disc’s ability to support axial loads, absorb shock, facilitate spinal mobility, and maintain vertebral stability.

Disc degeneration is marked by the breakdown of ECM components, including hyaluronic acid and major structural macromolecules such as aggrecan—a critical cartilage-specific proteoglycan that contributes to tissue hydration and biomechanical integrity [11].

The IVD is an avascular structure; nutrient supply is maintained through capillaries located in the outer AF [12] and via diffusion across the cartilaginous EPs [13]. Under physiological conditions, only the outer layers of the AF are innervated, primarily by sinuvertebral nerves and branches of the ventral spinal roots and gray rami communicantes. These nerves include both small and large fibers functioning as mechanoreceptors and nociceptors [14].

During disc degeneration, immune cell infiltration leads to the release of pro-inflammatory cytokines such as tumor necrosis factor-alpha (TNF-α), interleukins IL-1β, IL-6, and IL-17 [15,16], initiating and perpetuating a degenerative cascade [17]. In synergy with these cytokines, neurotrophic factors—including 1B4, nerve growth factor (NGF), calcitonin gene-related peptide (CGRP), brain-derived neurotrophic factor (BDNF), and neuropeptides such as substance P (SP)—along with S-100 proteins, have been implicated in the sensitization of nociceptive pathways and the generation of pain [18]. Even low concentrations of IL-1β (1 pg/mL) and NGF (≥0.1 ng/mL) can significantly upregulate NGF and SP expression in NP cells [19]. SP contributes to neovascularization within the degenerating IVD [20]. Prolonged exposure to SP has been shown to upregulate pro-inflammatory cytokine production via activation of the NF-κB signaling pathway, thereby accelerating disc degeneration and contributing to discogenic pain [21]. Moreover, SP-positive nerve fibers identified in degenerated discs are hypothesized to be associated with neoangiogenesis [22].

S-100 proteins represent a group of calcium-binding, acidic peptides specific to neural tissue, exhibiting molecular and charge heterogeneity but immunological similarity [23]. In the central nervous system, 85–90% of total S-100 protein is localized in astrocytes, 10–15% in neurons, and trace amounts in oligodendrocytes. These proteins are synthesized by glial cells and subsequently transported to neurons. Intracellularly, S-100 proteins are primarily localized in the cytoplasm, synaptic membranes, and chromatin, where they regulate processes such as proliferation, apoptosis, inflammation, migration, tissue repair, invasion, and immune signaling.

However, members of the S100 family are expressed in a variety of tissues and cell types beyond the nervous system. For instance, S100A4 is expressed in some tumor cells, T cells, neutrophils, and macrophages; S100A7 in epithelial cells; S100A8 and S100A9 in monocytes, granulocytes, keratinocytes, epithelial cells, and osteoclasts; S100A11 shows ubiquitous expression in many tissues; and S100B is found also in melanocytes, chondrocytes, adipocytes, myocytes, dendritic cells, and lymphocytes. Furthermore, S100B binds to and signals through the RAGE on chondrocytes. In this context, extracellular S100B acts as a pro-catabolic and pro-inflammatory mediator that promotes cartilage degradation and, as reported in the literature, contributes to the pathogenesis of osteoarthritis and rheumatoid arthritis [23]. These findings support the relevance of investigating S-100 expression in the context of IVD degeneration.

Given the increasing interest in the role of neurotrophic markers such as NGF and S-100 in the pathogenesis of DDD and associated LBP, further investigation is warranted.

The aim of this study was to evaluate the molecular and cellular mechanisms of pathological neo-innervation and ECM degradation in IVD degeneration in young adults with chronic discogenic LBP. We applied histological staining (hematoxylin and eosin (H&E), alizarin red, and trichrome) alongside immunohistochemical (IHC) analysis using established markers NGF, SP, S-100, and aggrecan.

## 2. Results

### 2.1. ECM Degradation in the IVD (Calcification)

According to Sive’s criteria, the histological degeneration score of the IVD was significantly higher in the patient group compared to healthy controls: 5.000 [3.000, 6.750] vs. 1.000 [0, 1.000], respectively (Mann-Whitney U test: *p* = 0.0001).

**Figure 1:**
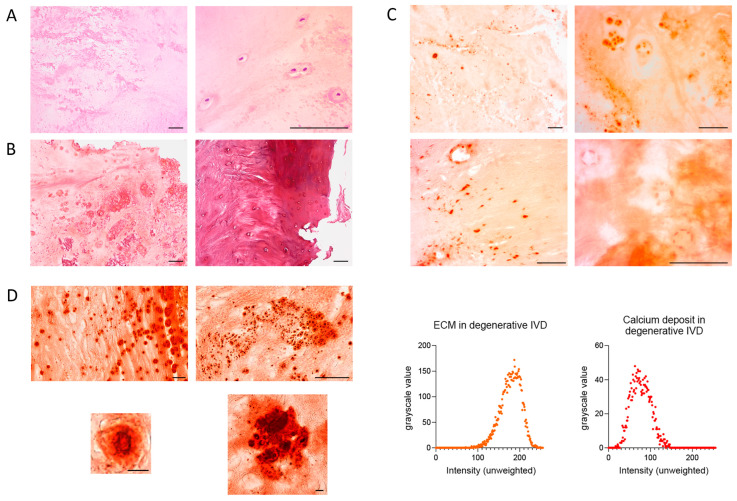
Calcification in intervertebral disc (IVD) tissue in patients with varying degrees of IVD degeneration. (**A**). Hematoxylin-eosin staining. (**B**). Trichrome staining: left-nucleus pulposus (NP); right-transition from annulus fibrosus to vertebral endplate. (**C**). Alizarin red staining: upper-NP; lower-annulus fibrosus (left) and hypertrophied chondrocyte clusters in the NP (right). Scale bar for (**A**–**C**): 100 µm. (**D**). Individual calcifications in IVD tissue stained with alizarin red, imaged using a histology scanner. Scale bar for top images: 50 µm; for bottom images: 15 µm. Comparison of grayscale intensity distribution between calcified areas and IVD extracellular matrix (ECM) using the 0–255 scale in ImageJ 1.52u, where 0 corresponds to pure black and 255 to pure white.

Calcifications were observed in 36.1% of patients with DDD, as revealed by H&E staining. These appeared as rarefied, dark-pink regions with rod-like or rounded morphology. In some areas, they coalesced into continuous zones with scalloped borders (Figure 1A). Trichrome staining confirmed these calcifications as pinkish-lilac areas, closely resembling those seen in H&E-stained sections (Figure 1B). Calcifications were more frequent in the NP and were often associated with fissures. Hyaline cartilage in the EPs stained claret red (Figure 1B).

Alizarin red staining of IVD samples was irregular and non-specific, appearing in both central and peripheral regions, particularly near hypertrophic chondrocytes, cell clusters, and intact chondrocytes. In acellular areas, staining varied from bright red to pale orange (Figure 1C). In hyaline cartilage of the EP, darker regions likely reflected denser, mineralized matrix. Chondrocyte nuclei stained darker than the ECM but lighter than the calcifications; cytoplasm had the same hue as the ECM. Nuclei of endothelial cells and fibroblasts in the AF also showed relatively dark staining (Figure 1C). Importantly, there was no evidence of peripheral or fissure-related staining artifacts.

Histological scanner images revealed calcifications with sharply defined black outlines, occasionally interrupted by fissures, enclosing bright red centers of greater intensity than the surrounding disc tissue. The core of smaller calcifications appeared brown and occupied ~80% of their total area (Figure 1D). Some calcifications were polygonal, with a length up to three times their width. The smallest measured approximately 1.7 µm. Larger calcifications often contained internal black striations. Based on grayscale analysis, the mean intensity of calcifications was 77.40 ± 22.76, whereas that of the ECM was 178.66 ± 24.23.

### 2.2. Aggrecan Loss

**Figure 2:**
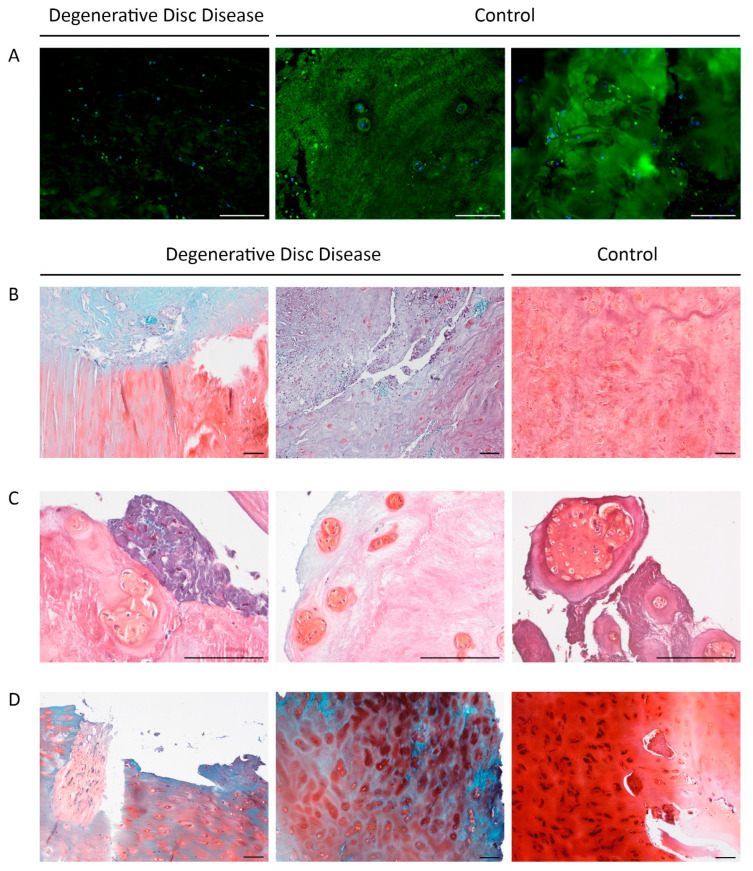
Extracellular matrix degradation in degenerative disc disease (DDD): reduced aggrecan content, erosive changes, and fissures. (**A**). Immunofluorescence staining in nucleus pulposus (NP) regions of DDD and control samples: aggrecan (green), nuclei counterstained with DAPI (blue). (**B**). Safranin O/Fast Green FCF staining. Safranin O stains cartilage glycosaminoglycans pink to red, while Fast Green FCF counterstains the background bluish green; nuclei appear purple to black. Left: transition zone between NP and annulus fibrosus (AF). Center and right: NP region. (**C**). Safranin O staining of degenerated NP with chondrocyte clusters. (**D**). Vertebral endplate in DDD patient; blue staining reflects degeneration. Black-stained chondrocytes on the right may point to pigment accumulation due to cell stress or apoptosis. Scale bar: 100 µm.

Sections stained for aggrecan exhibited markedly reduced fluorescence intensity in DDD patient samples compared to controls (Figure 2A). Pronounced aggrecan depletion was observed in 7 patients (19.4%; 3 females and 5 males; median age: Me = 33.00 [20.00, 36.00]), and correlated significantly with the duration of the current episode of LBP (r = 0.449; *p* = 0.031; median duration: = 6.00 [4.00, 12.00] weeks).

The area stained with Fast Green, indicating ECM pathology, also correlated with pain episode duration (r = 0.455; *p* = 0.029). Figure 2B–D show Safranin O staining of NP, AF, and EP. ECM staining ranged from light turquoise and pink to red-pink, violet, and blue. Chondrocyte clusters appeared darker and predominantly red, reflecting increased metabolic activity.

In DDD patients, the vertebral EPs exhibited erosive changes and fissures. ECM staining was predominantly blue, characteristic of hyaline cartilage loss. However, chondrocytes and the narrow pericellular matrix retained a carmine hue, suggesting preserved constitutive expression. In one DDD patient, chondrocytes stained black (Figure 2D, right panel), possibly due to pigment accumulation associated with cellular stress or apoptosis.

No statistically significant differences in the Safranin O-stained NP area were found between patient and control groups. Similarly, the two main Fast Green staining types-turquoise and blue-did not differ between groups, indicating substantial ECM heterogeneity even within cohorts.

Histological degeneration scores showed no significant correlation with age (r = 0.138, *p* = 0.5), reinforcing that the degeneration observed was pathological rather than age-related.

### 2.3. Expression of NGF, S-100, and SP in the IVD

**Figure 3:**
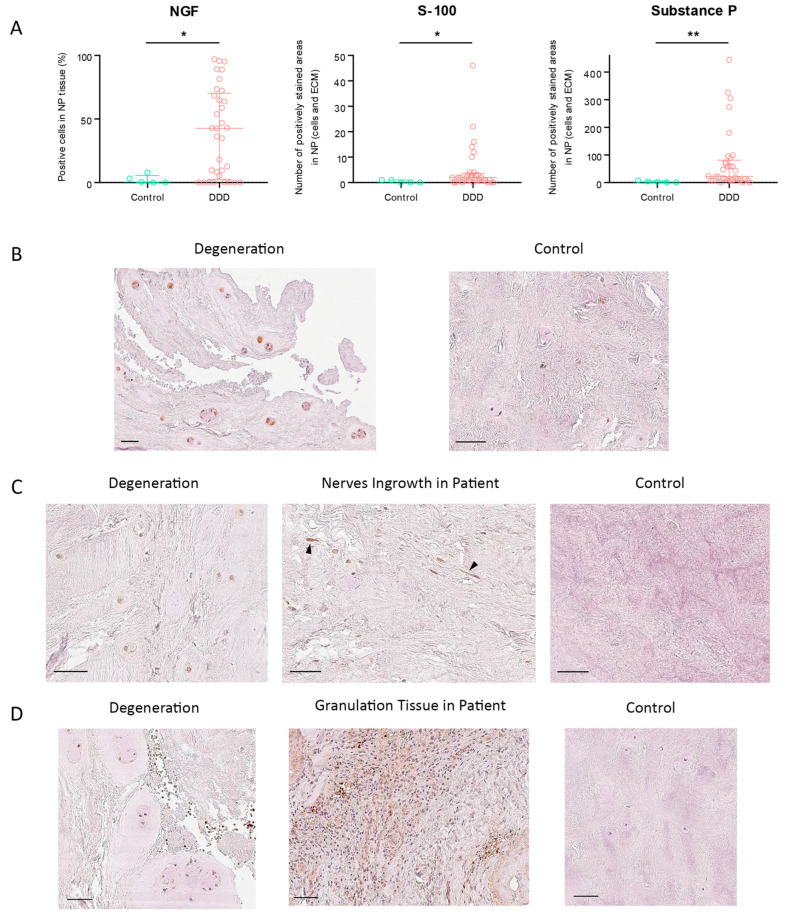
Immunohistochemistry (IHC) of NGF, S-100, and Substance P (SP) in NP cells of degenerative disc disease (DDD) patients and controls. IHC was performed using DAB chromogen and Gill’s hematoxylin counterstain. (**A**). Statistical comparison (Mann–Whitney U test). (**B**). NGF IHC. (**C**). S-100 IHC. Arrows indicate nerve ingrowth in patient tissue. (**D**). SP IHC. Scale bar: 50 µm. *: *p* < 0.05, **: *p* < 0.01. ECM—extracellular matrix, NP—nucleus pulposus.

The percentage of NGF-positive cells in NP tissue was significantly higher in DDD patients than in controls (*p* = 0.0287) (Figure 3A). In the control group, the highest observed NGF expression was 7.95%; notably, 8 DDD patients exhibited no detectable NGF immunoreactivity. Among all assessed markers, NGF showed the highest expression, reaching a maximum score of 3/3 (Figure 3B). NGF expression strongly correlated with SP levels (r = 0.785; *p* = 5.268 × 10^−9^), suggesting their potential co-involvement in nociceptive signaling and pain generation in DDD. Conversely, SP expression was virtually absent in controls.

One outlier patient presented an abnormally high number of S-100-positive cells (*n* = 380) and was excluded from further analysis. S-100 expression in NP cells and ECM was significantly elevated in DDD patients compared to controls (*p* = 0.0312) (Figure 3B), with a staining score of 1/3 (Figure 3C). Free nerve endings were observed in 5 DDD patients, while none were present in controls, indicating pathological neo-innervation of the IVD.

SP expression showed the greatest differential between degenerated and healthy discs among all tested markers, both in NP cells and ECM (*p* = 0.0039) (Figure 3C), with a moderate staining intensity of 2/3 (Figure 3D). Although SP expression correlated most closely with S-100, the correlation was weak and not statistically significant (r = 0.281; *p* = 0.088).

**Figure 4:**
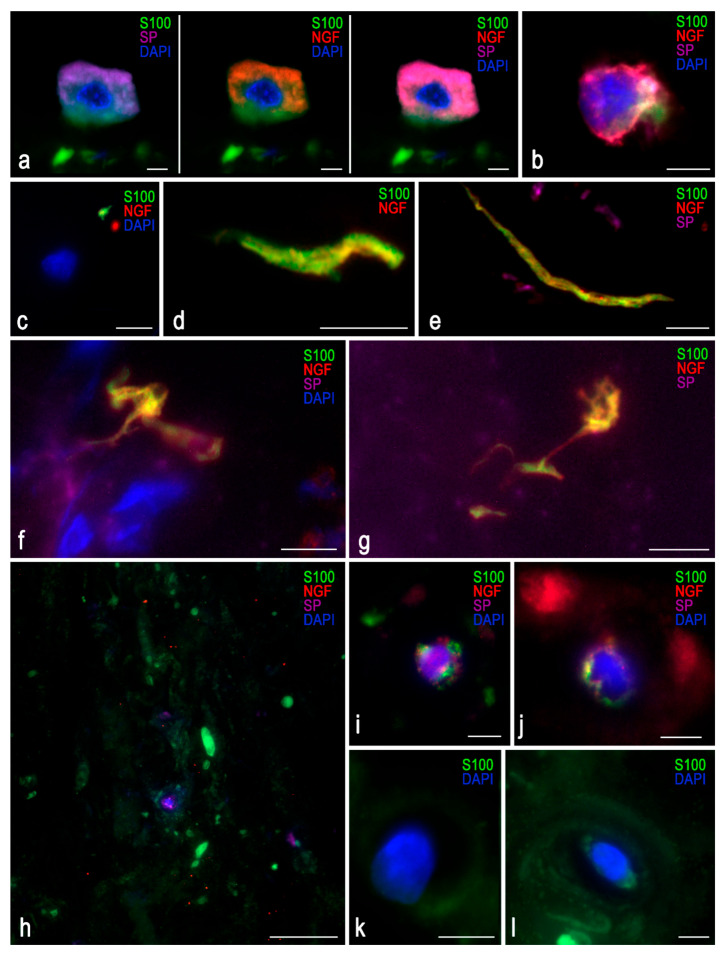
Triple immunofluorescence for S-100 (green), NGF (red), and Substance P (SP, violet) in intervertebral disc (IVD) tissue. (**a**–**g**): Samples from degenerative disc disease (DDD) patients. Images show a presumptive mast cell (**a**) and a lymphocyte (**b**). A nerve fiber is shown growing near a chondrocyte, with NGF localized nearby; the chondrocyte’s fluorescence is restricted to its nucleus (**c**). A nerve fiber lacking SP expression (**d**). Nerve fibers in topographical proximity to SP (**e**–**g**). (**h**–**l**): Control samples. Non-specific staining was observed in areas of tissue densification, particularly for S-100. Only a few chondrocytes demonstrated visible NGF and SP staining (**h**–**j**). Scale bar: 5 µm.

Triple immunofluorescence was used to evaluate marker co-localization (Figure 4). DDD samples showed a dominant violet background from SP staining, which did not overlap with the S-100 signal, ruling out autofluorescence. Autofluorescence, most notable in the S-100 detection channel, was minimized to preserve tissue detail.

A cell with morphological characteristics resembling those of a mast cell was identified within an IVD fissure (Figure 4a); however, further staining for tryptase or chymase was not performed. A lymphocyte was also presumptively detected (Figure 4b). NGF was detected as distinct puncta (up to 5 µm) even in control tissues. In DDD samples, NGF localized near S-100-positive zones and close to nerve endings, with a mean marker separation of 0.5 µm (Figure 4c–g). Figure 4h–j show isolated chondrocytes with constitutive expression of all three markers.

### 2.4. Correlation Analysis

**Figure 5:**
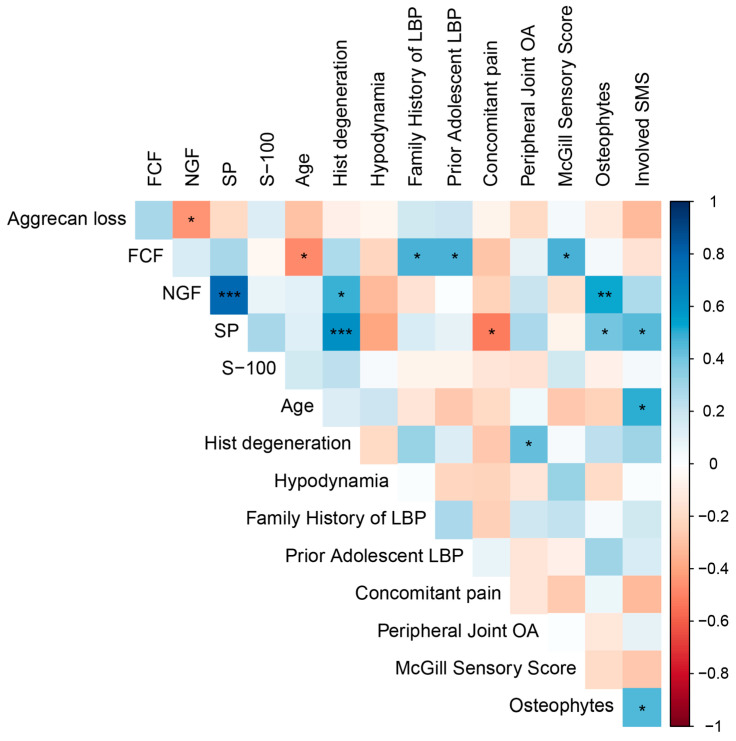
Correlation between clinical-imaging findings and histochemical/immunohistochemical markers (Spearman’s correlation coefficient). Note: FCF—Fast Green FCF stain; SP—substance P; LBP—low back pain; OA—osteoarthritis; SMS—spinal motion segment. Statistical significance in the correlation matrix is denoted as *p* ≤ 0.05 (*), *p* < 0.01 (**), and *p* < 0.001 (***); lack of an asterisk denotes a non-significant correlation (*p* > 0.05).

We observed a positive correlation between the expression levels of NGF and SP and the severity of histological disc degeneration (r = 0.467, *p* = 0.010 and r = 0.611, *p* = 0.0006, respectively) (Figure 5), indicating a pathogenetic link between neuroinflammation and cartilage degeneration that is independent of age. Interestingly, an inverse correlation between disc degeneration and age within the DDD group (r = −0.471, *p* = 0.0151) underscores the pathological rather than purely age-related nature of the degenerative process.

Expression levels of both NGF and SP also positively correlated with the presence of 3–5 marginal osteophytes at the lumbar level (r = 0.526, *p* = 0.0083 and r = 0.409, *p* = 0.0472, respectively), suggesting an association between spinal spondylosis, disc neo-innervation, and nociceptive back pain. Furthermore, the number of spinal motion segments affected, as determined by magnetic resonance imaging (MRI) (disc herniation combined with osteophytosis and facet joint osteoarthritis), also correlated with SP expression (r = 0.446, *p* = 0.0291).

A history of back pain during adolescence was linked to more severe adult disc degeneration and the presence of calcification (r = 0.476, *p* = 0.0216), potentially reflecting a genetic predisposition to early-onset axial skeletal degeneration. These individuals also exhibited a positive family history of LBP (r = 0.474, *p* = 0.0222).

A moderate negative correlation was observed between smoking and aggrecan loss (r = −0.502, *p* = 0.0147), which requires clarification in studies with larger numbers of participants. In contrast, a positive correlation was found with pain intensity (visual analog scale score) (r = 0.567, *p* = 0.0048), while pain episode duration was negatively associated with smoking (r = −0.418, *p* = 0.0474). Furthermore, antalgic posture was more frequently reported in smokers (r = 0.481, *p* = 0.0202).

We also investigated the associations of obesity with clinical and behavioral variables. Notably, obesity showed a significant positive correlation with the presence of antalgic posture (r = 0.471, *p* = 0.0232). Moreover, the degree of obesity correlated positively with the number of cigarettes smoked per day (r = 0.443, *p* = 0.0343).

## 3. Discussion

Aggrecan degradation is a hallmark of degenerative musculoskeletal disorders and has been extensively studied in osteoarthritis [24]. Rutges JP et al. proposed a grading system for NP degeneration based on Safranin O staining: intense red staining (F0) progressively diminishes, transitioning through a mixed red-green appearance (F1), to weak Fast Green-dominated staining (F2) [25]. In a murine model of EP sclerosis, 20-month-old mice exhibited dark blue Fast Green staining, in contrast to the turquoise coloration observed in 3-month-old mice [26]. In our study, aggrecan staining in human IVD tissue appeared pink, resembling patterns reported in animal models [27]. No correlation was found between Fast Green staining intensity, used as a marker of histological degeneration, and patient age. The relationship between aggrecan loss and disc calcification remains poorly understood in both animal models and human tissues.

The presence of nerve fibers within the NP is a known feature of IVD degeneration and is associated with discogenic back pain. Neo-innervation may take several forms, including free nerve endings, Ruffini corpuscles, Aδ- and C-fibers, and perivascular nerves [14]. Our findings demonstrated high levels of NGF expression within the NP and surrounding ECM, indicating local neo-innervation during IVD degeneration. Co-localization of NGF and SP further supports a mechanistic link between nerve growth and pain signaling. SP was diffusely expressed in the ECM of degenerated discs, particularly along elongated, branching nerve fibers. Previous research has shown that anti-NGFβ treatment can reduce axonal outgrowth and SP expression in NP tissue, though it has little impact on the AF [28].

While the role of S-100 proteins in IVD chondrocytes remains insufficiently characterized, our observations suggest their potential involvement in DDD. The absence of correlation between histological degeneration and S-100 expression may reflect sampling variability during surgery, as innervation within degenerated discs is not uniformly distributed throughout the NP. Recent studies have highlighted the pathological relevance of specific S100 proteins in DDD. In particular, S100A9 is markedly upregulated in degenerated NP tissue and promotes NP cell apoptosis, ECM degradation, and inflammation [29]. Together with S100A8, it acts as an alarmin, inducing a catabolic and pro-inflammatory environment by stimulating MMP-3/13, TNF-α, IL-1, IL-8, IL-6, and ADAMTS-4, while suppressing aggrecan and collagen II synthesis via TLR4-mediated activation of the NF-κB signaling pathway [30]. Inhibition of S100A9 has been shown to alleviate both disc degeneration and associated inflammatory pain, underscoring its potential as a therapeutic target in DDD.

An underexplored degenerative phenotype is disc calcification, characterized by calcium phosphate accumulation and extensive cell death within the IVD [31]. Novais EJ et al. identified increased susceptibility to degeneration at C5–6, T6–7, and L4–5 levels, though calcification patterns varied across spinal levels [32]. Whether this reflects biomechanical stress or a generalized degenerative cascade remains unclear. Disc calcification has also been linked to systemic conditions such as chondrocalcinosis, pseudogout, ankylosing spondylitis, and hyperparathyroidism, pointing to potential metabolic or inflammatory contributions. While MRI cannot directly detect microcalcifications, high T1 signal intensity may suggest their presence, or reflect fatty infiltration [33,34,35]. Population-based studies report disc calcification in up to 6% of patients with DDD, with the AF being affected in 63% of cases across all age groups [36].

Histologically, calcified human and animal discs show increased levels of calcium, inorganic phosphate, TNAP, and collagen type X. The mechanism of calcification differs by disc region: in AF and EP, it represents heterotopic calcification regulated by pyrophosphate metabolism (PPi), while NP calcification is dystrophic-characterized by amorphous calcium crystal deposition following cell necrosis, independent of PPi signaling [31,37]. The molecular mechanisms driving calcification in DDD warrant further investigation. In our cohort, ectopic cartilage calcification was identified in one-third of DDD patients.

According to Rutges JP et al., DDD is accompanied by increased expression of osteoprotegerin (OPG), collagen type X, and Runx2, with OPG demonstrating the strongest correlation with degeneration in both NP and AF regions. These molecular changes resemble those observed in osteoarthritis, including hypertrophic differentiation and calcification, which are visible on micro-CT imaging [38]. OPG inhibits bone resorption by binding RANKL and preventing its interaction with RANK on osteoclasts [39]. In a rat model of disc degeneration, IL-1β stimulation increased RANKL mRNA expression; however, RANKL exerted catabolic effects only in the presence of IL-1β [40]. Subsequent investigations conducted by the aforementioned research team showed that IL-1β induced both RANKL and OPG expression in NP and AF cells from DDD patients, though the RANKL/OPG ratio remained unchanged. RANKL and OPG mRNA levels were significantly higher in NP cells than in AF cells [41].

Mast cells are known to release a wide range of inflammatory and angiogenic mediators upon activation, including VEGF, FGF-2, tryptase, chymase, IL-8, TGF-β, TNF, and NGF [42]. In addition, mast cell-derived exosomes can stimulate collagen synthesis and proline hydroxylation in fibroblasts, mimicking TGF-β effects and contributing to fibrotic remodeling. The presence of mast cells in NP tissue may exacerbate inflammation and ECM alterations, thereby promoting degeneration, although their precise role in DDD pathogenesis requires further investigation.

Our earlier publications detailed the expression of pro-inflammatory markers and angiogenic factors in the same patient cohort, using both molecular genetic analysis and immunohistochemistry [15,16]. As noted, the current manuscript is a continuation of this work, with a specific focus on the neural and glial components of disc degeneration. Accordingly, cytokine profiling remains an integral part of our broader investigation into the pathogenesis of DDD.

## 4. Materials and Methods

### 4.1. Patient Samples

This study was conducted at the Pirogov Russian National Research Medical University between 2023 and 2024 and was approved by the local ethics committee (protocol No. 208, dated 17 May 2021). Informed consent was obtained from all participants. Patients were consecutively recruited from the Neurosurgical Department of N.I. Pirogov State Clinical Hospital No. 1 in Moscow. All 36 patients were scheduled to undergo microdiscectomy due to progressive LBP and/or neurological symptoms caused by IVD herniation.

The median age was Me = 36.00 [31.00, 42.50] years; 36.11% were female and 63.89% male. The average intensity of back pain on a visual analog scale was 71 [61.00, 90.00] mm. In 87% of the cases, symptoms had a chronic course lasting from 2 to 10 years (Table 1). Patients with infectious, traumatic, oncological, or rheumatic spinal lesions were excluded from the study.

According to MRI of the lumbar spine, the stage of disc degeneration at the level of extrusion ranged from grade III to grade V per the Pfirrmann classification, corresponding to advanced (grade III), severe (grade IV), and irreversible (grade V). Degeneration was characterized by marked NP disorganization and dehydration. MRI revealed erosive EP damage in 58.33% of patients, marginal vertebral osteophytes in 66.67%, and reactive changes in the adjacent vertebral bodies (Modic changes) in 56.52% of patients (60% Type I, 40% Type II). Facet joint osteoarthritis was present in 91.67% of cases.

Disc tissue samples were obtained during discectomy. Under general endotracheal anesthesia and with the patient in the lateral position, a ~3 cm skin and soft tissue incision was made at the interspinous space of the lower lumbar vertebrae. This was followed by careful skeletonization of the adjacent vertebral arches and interspinous ligaments, interlaminectomy, and flavotomy. The herniated disc fragment was removed en bloc under a surgical microscope, and the disc cavity was curetted. The surgical wound was closed in layers. The specimens appeared as whitish, dense, cartilage-like tissue and were fixed in 10% buffered formalin (pH = 7.4) (Biovitrum, #B06-003, ErgoProduction LLC, Saint Petersburg, Russia).

Control samples were obtained from five previously healthy individuals (median age Me = 40.00 [38.50, 42.00] years, all male) who sustained acute motor vehicle trauma and required spinal stabilization. IVD segments (L4–L5, L5–S1) were collected within 1 h post-injury. All specimens contained NP tissue, which was the focus of further analysis.

### 4.2. Histochemical Staining

To assess degenerative changes beyond the Pfirrmann grading, we performed H&E, alizarin red, and trichrome staining.

Immediately after discectomy, IVD specimens were fixed in 10% buffered formalin (pH = 7.4) (Biovitrum, #B06-003, ErgoProduction LLC, Saint Petersburg, Russia). The samples were embedded in paraffin, sectioned at 3 µm, and mounted on glass slides. Staining with Gill’s hematoxylin (Biovitrum, #05-003) and eosin (Biovitrum, #05-010/LN) was performed according to a standard protocol. The protocol included two changes of xylene (3 min each), followed by immersion in absolute isopropanol (99.7%) for 6 min. The slides were then passed through 96% ethanol for 2 min, 80% ethanol for 2 min, and distilled water for 2 min. Hematoxylin staining was performed for 3 min, followed by rinsing in tap water for 1 min to enhance nuclear contrast. After staining with eosin for 1 min, the slides were differentiated in 96% ethanol by gentle dipping for 1 min, then placed in absolute isopropanol (99.7%) for 4 min, and finally cleared in two changes of xylene (3 min each).

For calcium identification, alizarin red S staining (#A5533-25G, Sigma-Aldrich, Burlington, MA, USA) was used: 2 g of the dye was dissolved in 100 mL of distilled water. Sections were stained for 3 min, dehydrated through three changes of absolute isopropanol, and coverslipped using the Tissue-Tek Film Automated Coverslipper (Sakura Finetek USA, Torrance, CA, USA).

Trichrome staining was used to distinguish calcified regions from cartilage. After deparaffinization and hydration in distilled water, slides were incubated in Weigert’s iron hematoxylin (prepared by mixing equal parts of Weigert’s Hematoxylin A (#E-001/1000, Labiko, Saint Petersburg, Russia) and B (#E-002/1000, Labiko)) for 5 min, rinsed in distilled water, and immersed for 2 s in 1% aqueous acetic acid. After additional rinsing, the slides were sequentially treated with 0.05% Fast Green FCF C.I. 42053 (#2353-45-9, Sigma-Aldrich) for 1 min, 1% acetic acid for 30 s, and 0.1% Safranin O C.I. 50240 (#477-73-6, Sigma-Aldrich) for 10 min. After dehydration in three changes of absolute isopropanol and subsequent xylene clearing, the sections were coverslipped using the Tissue-Tek Film Automated Coverslipper (Sakura Finetek, Torrance, CA, USA).

### 4.3. Immunostaining

All staining protocols were performed in accordance with standardized procedures for histochemical and IHC methods, including multiplex IHC staining, as described in Buchwalow & Boecker [43] and Buchwalow et al. [44]. Primary antibodies were used at optimized dilutions based on titration experiments, regardless of manufacturer recommendations. Specificity of IHC staining was assessed using both negative and positive controls. For negative controls, primary antibodies were replaced with corresponding non-immune IgG from the same species and at the same concentration. For positive controls, tissues known to express the target antigen were used to verify antibody specificity [44]. In addition, to rule out autofluorescence when using fluorescent dyes, sections were incubated in PBS and examined under a ZEISS Imager.Z2 fluorescence microscope (Carl Zeiss Vision, Jena, Germany). The spectral range of endogenous autofluorescence did not overlap with that of the fluorophores conjugated to the secondary antibodies.

IHC staining of IVD tissue was performed to detect aggrecan (#PAB908Hu01), SP (#PAA393Hu01), NGF (#PAA105Hu01) (all at 1:150 dilution, rabbit polyclonal antibodies, Cloud-Clone Corp., Katy, TX, USA), and S-100 (1:150, mouse monoclonal antibodies, #MAA012Hu22, Cloud-Clone Corp.).

For chromogenic detection, we used HRP-conjugated secondary antibodies: AmpliStain™ anti-Mouse 1-Step HRP (#AS-M1-HRP, SDT GmbH, Baesweiler, Germany) for mouse primary antibodies and AmpliStain™ anti-Rabbit 1-Step HRP (#AS-R1-HRP, SDT GmbH) for rabbit primary antibodies. Color development was performed using the ImmPACT™ DAB Peroxidase Substrate Kit (#SK-4105, Vector Laboratories, Burlingame, CA, USA) according to the manufacturer’s instructions.

Secondary antibodies for immunofluorescence included Goat Anti-Mouse IgG H&L (AlexaFluor^®^ 488, #ab150113-500, Abcam, Cambridge, MA, USA) for S-100, Goat Anti-Rabbit IgG H&L (AlexaFluor^®^ 594, #ab150080, Abcam) for NGF, and OPAL700 (from the OPAL fluorochrome series for the Mantra 2 Quantitative Pathology Imaging System, Akoya Biosciences, Marlborough, MA, USA) for SP. Cell nuclei were counterstained using Fluoroshield Mounting Medium with DAPI (#ab104139-20, Abcam). Sections were mounted using VECTASHIELD^®^ Antifade Mounting Medium (#H-1000, Vector Laboratories).

### 4.4. Morphometric Analysis

For light microscopy, an Olympus CX43 microscope with Olympus SC50 camera (Olympus Co., Tokyo, Japan) and ScanScope CS scanner (Leica Biosystems, Deer Park, IL, USA) were used. For immunofluorescence imaging, a ZEISS Axio Imager.Z2 microscope (Carl Zeiss Vision, Jena, Germany) was employed. Areas showing staining for all three markers in identical locations were considered artifacts and excluded from analysis. Image analysis was performed using QuPath software (v0.4.3), employing automated positive cell detection algorithms with predefined parameters, including color deconvolution and intensity thresholds. Expression levels were graded from 1 (weak) to 3 (strong).

The stage of degeneration was determined according to the Sive criteria [45], where a score of 0–3 was considered normal, 4–9 indicated degeneration, and 10–12 indicated severe degeneration.

### 4.5. Statistical Analysis

The Graph Prism 8.0.1 (GraphPad, La Jolla, CA, USA) was used for statistical analysis. The distributions were determined to be parametric by Shapiro-Wilk testing. All measurement data were expressed as median [Q1, Q3]. To evaluate correlations between clinical-imaging parameters and histological/IHC markers, Spearman’s rank correlation coefficient was used. The Mann-Whitney U test was used to compare quantitative data between the two groups. A *p*-value < 0.05 was considered statistically significant.

## 5. Conclusions

This study elucidates molecular and cellular mechanisms underlying IVD degeneration and discogenic pain. We demonstrate that aggrecan loss and tissue calcification are associated with pathological neo-innervation. The expression of NGF and SP in degenerated IVD highlights their critical role in the development of nociceptive pain. Although histological evaluation is not feasible for routine clinical use, analysis of tissue from indicated discectomy offers valuable insight into end-stage disc pathology in young adults. These data may support future research aimed at identifying potential non-invasive biomarkers and developing targeted therapies to modulate pain-related signaling pathways. Further research should focus on chondrocyte—ECM interactions to prevent IVD degeneration and to validate the clinical relevance of these molecular signatures.

## 6. Study Limitations

In contrast to the study by Rutges JP et al. [38], which utilized Alizarin Red and Mayer’s hematoxylin staining for degeneration grading, we applied an alternative protocol. This method is not suitable for routine pathological diagnosis of DDD because IVD samples are typically decalcified before sectioning to avoid artifacts and fissures. Additionally, our method makes it difficult to precisely identify chondrocytes or assess the number and composition of cellular clusters.

The control group included only five previously healthy individuals with traumatic injuries, which limits both the statistical power and generalizability of the findings. While obtaining healthy IVD tissue is ethically and practically challenging, the limited size and specific nature of the control cohort should be considered when interpreting between-group comparisons.

## Figures and Tables

**Table 1:** Clinical characteristics of patients based on imaging data (*n* = 36).

Parameters	Values
Male	23 (63.89%)
Female	13 (36.11%)
Intensity of LBP on a visual analogue scale, mm	71.00 [61.00, 90.00]
Duration of the latest pain episode, weeks	4.00 [3.00, 10.00]
Presence of LBP	36 (100%)
Duration of chronic LBP, years	5.00 [2.00, 10.00]
Number of exacerbations over the past 12 months	2.00 [1.00, 2.00]
Stage of DDD according to Pfirrmann classification	
L1-L2	2.00 [1.00, 2.00]
L2-L3	2.00 [1.00, 2.00]
L3-L4	2.00 [1.00, 3.00]
L4-L5	4.00 [3.00, 4.00]
L5-S1	4.00 [4.00, 4.75]
Pfirrmann grade of the operated disc level, *n* (%)	
2	1 (2.78%)
3	2 (5.56%)
4	22 (61.11%)
5	11 (30.55%)
Level of disc herniation, *n* (%)	
L4-L5, L5-S1	12 (33.33%)
L4-L5	4 (11.11%)
L5-S1	19 (52.78%)
Erosive changes of the EPs	21 (58.33%)
Marginal osteophytes of the vertebral bodies	16 (66.67%)
Facet joint osteoarthritis	33 (91.67%)
Modic changes (Types I and II), *n* (%)	20 (56.5%)
Type I	12 (60%)
Type II	8 (40%)

Notes: LBP—low back pain; EPs—vertebral endplates adjacent to the intervertebral disc at the level of herniation.

## Data Availability

The datasets used and/or analyzed during the current study are available from the corresponding author on reasonable request.
